# Superconductive MgB_2_ Intercalated Muscovite with Dynamically Tunable Stress

**DOI:** 10.1021/acsomega.4c05303

**Published:** 2024-09-10

**Authors:** Shu-Hua Kuo, Yi-Cheng Chen, Yu-Chieh Wang, Wan-Zhen Hsieh, Ching-Yu Chiang, Cheng-Maw Cheng, Lu-Hsing Chen, Kuo-Ping Chen, Yu-Hao Tu, Jiunn-Yuan Lin, Ying-Hao Chu

**Affiliations:** † Department of Materials Science and Engineering, National Tsing Hua University, Hsinchu 300044, Taiwan; ‡ Institute of Physics, National Yang Ming Chiao Tung University, Hsinchu 300093, Taiwan; § National Synchrotron Radiation Research Center, Hsinchu 300092, Taiwan; ∥ Institute of Photonics Technologies, National Tsing Hua University, Hsinchu 300044, Taiwan; ⊥ College of Semiconductor Research, National Tsing Hua University, Hsinchu 300044, Taiwan

## Abstract

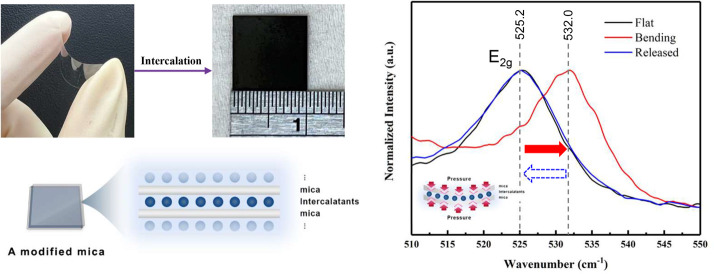

In this study, we utilized a stress-sensitive superconductor MgB_2_ in combination with a flexible muscovite, a layered silicate, to demonstrate that materials in a reduced-dimension environment could be influenced by external strain. MgB_2_ nanocrystals were inserted into the muscovite interlayers using gas phase intercalation, creating a two-dimensional cavity-like structure. Several experiments confirmed that the cavity-induced static pressure from the intercalation effect and the external dynamic bending effect can affect the physical properties of MgB_2_. The results of analyzing the changes in superconducting critical temperature (*T*_c_) indicate that the dynamic bending effect corresponds to an applied pressure of approximately 1.2 GPa. This method demonstrates that muscovite intercalation serves as a versatile platform for evaluating the stress effects on functional materials in reduced dimensions under ambient conditions.

## Introduction

Studying materials under high pressure is crucial in various scientific and industrial applications, with implications for earth sciences, materials science, physics, and chemistry.^1^ High pressure can significantly affect the physical properties of materials in several ways, for instance, phase transitions, density and volume, mechanical properties, electronic properties, optical properties, chemical reactivity, magnetic properties, and thermal properties.^2^ Exploring the fundamental aspects of materials under high pressure helps us understand their behaviors and potential applications. Hence, the diamond anvil cell (DAC) has been widely utilized for applying hydrostatic pressure in a variety of experiments.^3,4^ The components of a DAC are two opposing diamonds, and force is applied to the back of the diamonds, typically via a mechanical or hydraulic mechanism. DACs can be combined with analytical instruments used in various research fields to study the physical properties under external pressure. However, the size of the anvils is effectively limited due to the exponential increase in costs. Furthermore, it is difficult to use in practical applications. Herein, we offer an alternative cost-effective option for applying pressure to the sample by intercalating guest species into the interlayer spaces of muscovite. The size can be customized (from a few mm^2^ to cm^2^), which is available for almost every analytical instrument. Additionally, it can be transferred easily to different measurement setups without the need for a specific holder or configuration^1^ and can be used under ambient conditions.

**Figure 1:**
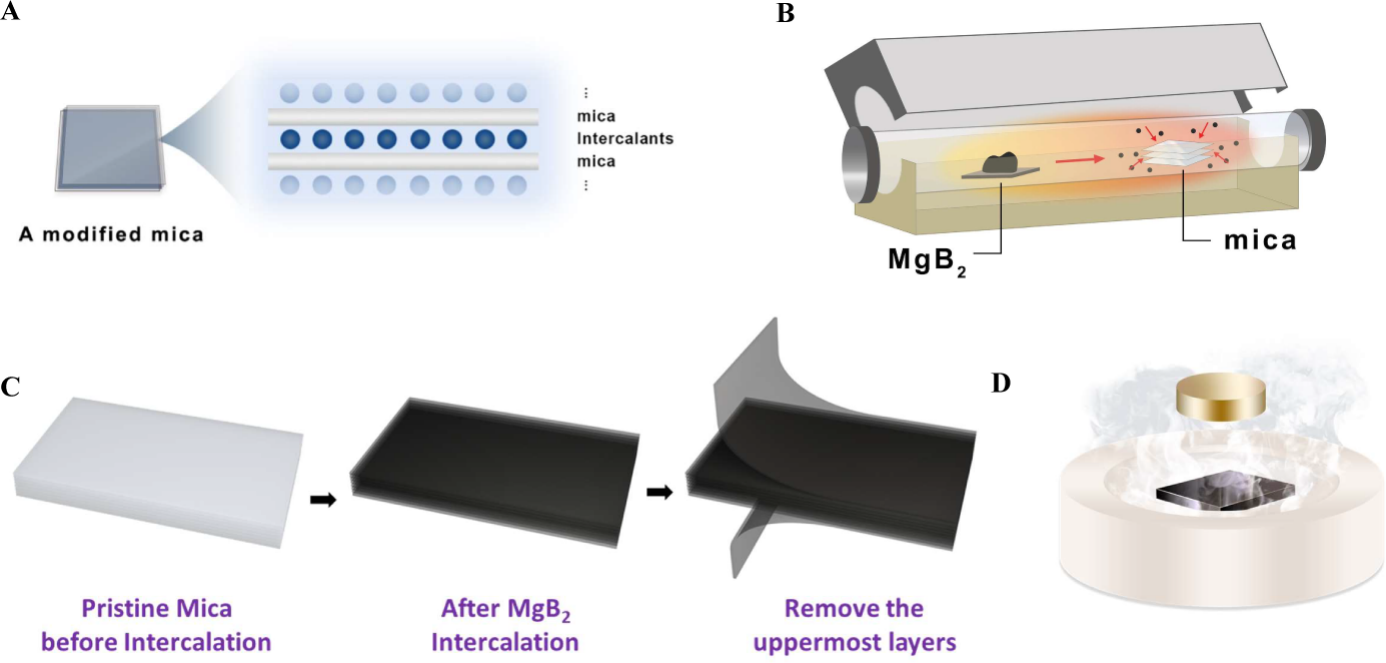
The schematic of the fabrication of intercalated muscovite. (A) The intercalants were intercalated in the interlayer of muscovite. (B) The setup of gas-phase intercalation. (C) The process flow of intercalated muscovite fabrication. (D) The illustration of the Meissner effect of the superconductor.

Synthetic muscovite with the formula KMg_3_(AlSi_3_O_10_)F_2_ is one of the layered silicates from the mica family, featuring a large dielectric constant (∼10) and a wide direct band gap (5.1 eV).^5^ Muscovite can be easily mechanically exfoliated down to a few single layers. Its interlayers, with vast lateral dimensions and interlayer spaces in a reduced volume, covalently bond atomic lattices via weak van der Waals interactions (Figure 1A). This offers various foreign species an opportunity to be inserted into interlayer spaces through intercalation processes without disrupting the in-plane covalent bonds. Moreover, muscovite is chemically inert, benefiting intercalants with a stable environment during and after the intercalation.^6^ The wide transparency of muscovite provides clear windows for optical measurements,^7^ allowing for the transmission of infrared, visible, and ultraviolet radiation (<5 eV) necessary for optical spectroscopy. Moreover, the muscovite interlayer can exert an intrinsic static pressure on the intercalants after an intercalation process. Given the inherent flexibility of muscovite, mechanical bending creates dynamic stress on the system. Bending muscovite with a serial radius further applies tunable external pressure onto intercalants, which can serve as an alternative system to DAC.

In this study, magnesium diboride (MgB_2_) is an example of intercalation fabrication and a high-pressure study. MgB_2_ is an intermetallic superconductor with an extraordinarily high critical temperature (*T*_c_) of ∼39 K.^8^ The simple binary boride crystallization with a simple hexagonal structure featuring outstanding *T*_c_ in an AlB_2_-type structure^9^ surprised the condensed matter field. It has generated considerable interest among theoretical and experimental researchers worldwide. Unlike cuprate superconductors, MgB_2_ exhibits superior transport properties,^10^ critical current density^11,12^ lower anisotropy,^13^ and grain boundaries that are transparent to current flow,^14^ making it an excellent candidate for various applications. MgB_2_ is a BCS type II phonon-mediated superconductor, which reduces the *T*_*c*_ under external pressure since the change of phonon frequency and charge transfer result in disordering and the reduction of the density of state.^15^

Superconductors confined to low dimensions exhibit remarkable physical properties that differ significantly from those of their bulk counterparts. This characteristic has been studied in previous research on intercalation, such as graphite intercalated compounds (GICs)^16^ and alkali-intercalated FeSe-based superconductors.^17,18^ These studies introduce guest intercalant between the host superconducting layers, aiming to modify the electronic structure of the host superconductor. This approach modifies the electronic structure of the host material through charge doping, structural distortions, and Fermi surface reconstruction. For instance, intercalating FeSe enhances its two-dimensionality and electron doping, potentially leading to higher *T*_c_.^19^ Our research presents an alternative approach by introducing the superconducting material (MgB_2_) as the guest intercalant within a host material (muscovite). The natural van der Waals gap between the muscovite interlayer can be viewed as a confined 2D cavity and provides the reduced dimension for exploring novel physical properties under ambient conditions

## Results and Discussion

The intercalation process can be categorized into two methods through the liquid phase or the gas phase. The liquid phase method is suitable for guest species such as transition metals with high melting temperatures. Conversely, the gas phase approach is typically favored for guest species that exhibit a high vapor pressure. Due to the tendency of MgB_2_ to oxidize into MgO upon exposure to water, we chose the gas phase approach, as illustrated in Figure 1B. MgB_2_ and muscovite are positioned at opposite ends within a quartz tube, allowing the MgB_2_ powder to transfer and intercalate into the muscovite under appropriate thermodynamic conditions. As depicted in Figure 1C, the initially transparent, insulating muscovite transforms into a black MgB_2_–muscovite composite after intercalation (Figure 1D). The process details can be found in the Methods section. To ensure that no residual MgB_2_ guest species on the surface interfere with subsequent physical properties measurements, we removed the muscovite surface layer.

**Figure 2:**
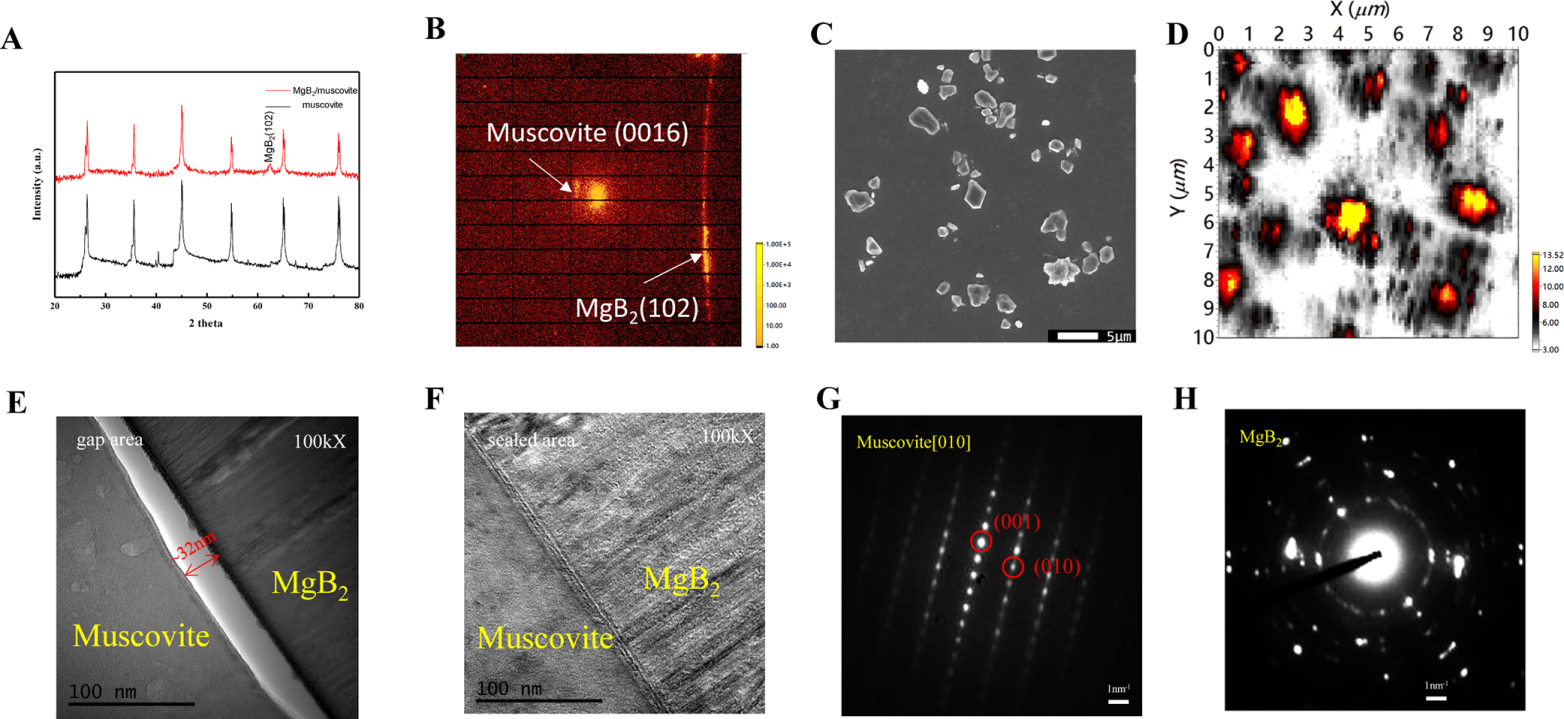
The structural characterization of the MgB_2_–muscovite composite. (**A**) The theta–2theta scan. (**B**) The Laue diffraction pattern. (**C**) The selected area of the X-ray nanodiffraction. (**D**) The intensity mapping of the MgB_2_(102) diffraction peak. (**E**) The gap area between the MgB_2_/muscovite interface. (**F**) The sealed area between the MgB_2_/muscovite interface. (**G**) The diffraction pattern of muscovite. (**H**) The diffraction pattern of the MgB_2_ crystal.

Initially, a series of structural characterizations were conducted on the MgB_2_–muscovite composite to understand the distribution and crystalline form of MgB_2_ within the muscovite interlayers. The theta–2theta measurements (Figure 2A) reveal a distinct MgB_2_(102) peak without evidence of other crystalline orientations or impure phases, such as MgO. However, MgB_2_ possesses a hexagonal structure, and muscovite is pseudohexagonal, which makes the growth orientation of MgB_2_(102) crystals slightly contradictory. Therefore, synchrotron radiation X-ray nanodiffraction was employed for selected area diffraction.^20^ Nanodiffraction patterns clearly show the diffraction spots of MgB_2_(102) and muscovite(0016) in Figure 2B. To further verify the crystal characteristics observed under SEM (Figure 2C), the diffraction peak of MgB_2_(102) on the area detector (Figure S1A) was included as the region of interest (ROI) for selected area X-ray nanodiffraction. The intensity mapping (Figure 2D) of the MgB_2_ intercalant reveals areas with high diffraction signal intensity (yellow-orange) that correspond well to the distribution of crystals in the SEM images. The darker regions do not indicate poor crystallinity but a tilting χ angle of MgB_2_(102) between several muscovite (00l) layers. It was observed (Figure S1B) that each grain exhibits a degree of tilt, leading to variations in signal intensity across the mapping (Figure S1C). A cross-sectional TEM analysis was conducted to gain further insight into the detailed structural information. Figure 2E,F show two distinct regions at the MgB_2_ crystal-muscovite interface: gap areas and sealed areas. The 32 nm thick space formed by these gaps delivers the reason for the variation of the χ angle in MgB_2_ nanocrystals. Clear diffraction patterns in Figure 2G,H demonstrate the high crystallinity of muscovite and the MgB_2_ intercalant.

**Figure 3:**
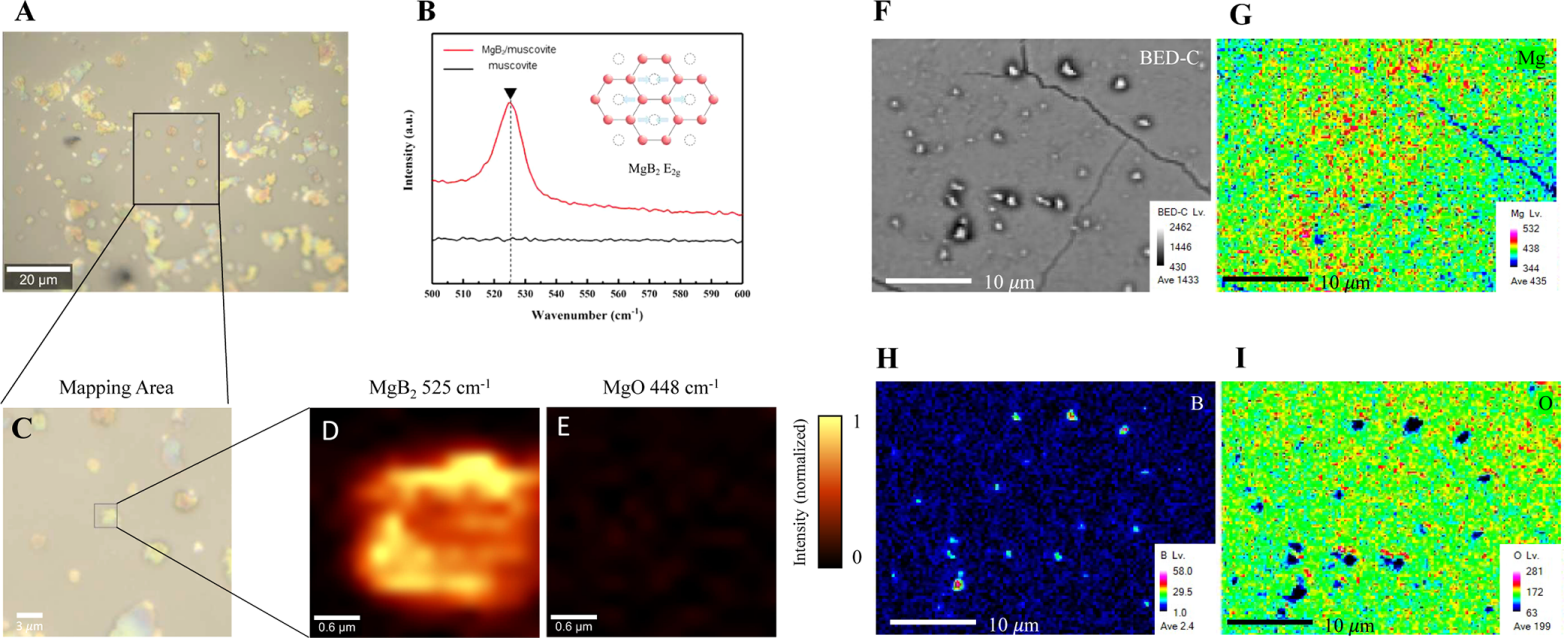
Spatial-resolution Raman spectra and elemental-sensitive EPMA characterization. (A) The distribution of MgB_2_ crystal under optical microscopy. (B) Raman spectra of MgB_2_/muscovite. (C) The selected mapping area of the Raman measurement. (D) The intensity mapping of the MgB_2_*E*_2g_ peak. (E) The intensity mapping of MgO indicated no Raman signal. (F) The scanning area morphology of EPMA mapping. The distributions of (G) Mg, (H) B, and (I) O.

In order to obtain information on sample quality and elemental composition, we utilized Raman spectroscopy and an electron probe microanalyzer (EPMA). Under the view of an optical microscope (Figure 3A), we can observe a lot of MgB_2_ crystals distributed in the muscovite. To characterize these crystals, a distinct *E*_2g_ mode of MgB_2_ can be observed in the Raman spectra (Figure 3B), where the *E*_2g_ mode appears at 525 cm^–1^. The small Lorentzian line width indicates a high sample quality, consistent with the calculations.^21^ The full-width half-maximum (fwhm) of the MgB_2_*E*_2g_ mode is 9.02 cm^–1^. Since *E*_2g_ is the only active mode for MgB_2_,^22^ we did not observe other optical modes (Γ = *B*_1g_ + *E*_2g_ + *A*_2u_ + *E*_2u_) and differences between the samples. Figure 3C shows the selected mapping area of the Raman measurement for MgB_2_ and MgO to evaluate the purity of the MgB_2_–muscovite composites. It suggests a strong intensity of the MgB_2_ crystal (Figure 3D) and an insignificant signal of MgO in Figure 3E, which implies the high purity of sample quality after the intercalation process. Since the boron atom (*Z* = 5) is too light to be detected by general X-ray photoelectron spectroscopy (XPS) and energy-dispersive X-ray spectroscopy (EDS), with no sufficient energy resolution for peaks of a boron atom, we then introduced EPMA to analyze the boron signal through its technical application of wavelength-dispersive X-ray spectroscopy (WDS). The qualitative elementary composition distribution is observed from the EPMA mapping result (Figure 3F–I). Figure 3F shows the scanning area morphology and the distribution contents of magnesium (Figure 3G), boron (Figure 3H), and oxygen (Figure 3I). The rainbow color map represents the relative quantity, where blueshift tends to indicated a lower amount, and redshift means a higher amount. It is clear that Mg and B distribute roughly in the same area, yet O lacks quantity in the same crystal, which supports the excellent purity of MgB_2_ in MgB_2_–muscovite composites. Moreover, the signals of Mg and O, which are generally distributed in the sample, primarily originate from the synthetic muscovite (KMg_3_(AlSi_3_O_10_)F_2_), mainly composed of SiO_2_, Al_2_O_3_, K_2_O, MgO, and other trace elements.

**Figure 4:**
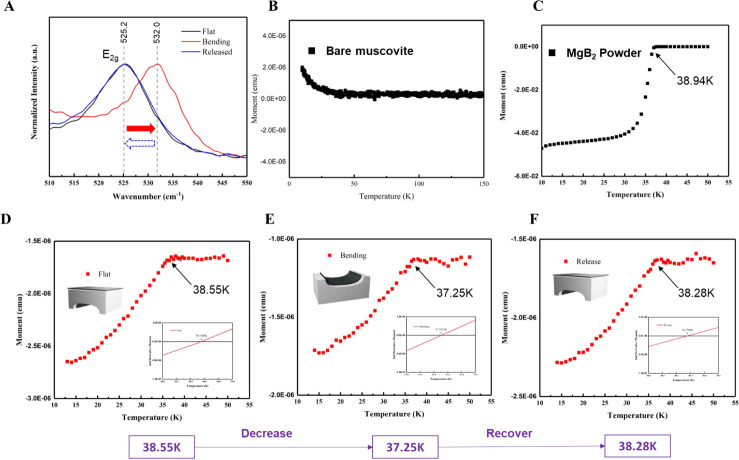
Physical properties under pressure. (A) The Raman spectra undergo a bending process. The M-T curves (B) of bare muscovite, (C) of pure MgB_2_ powder, (D) of the pristine state, (E) under the bending state, and (F) of the release state.

After characterizations of structural and chemical information, we investigated the superconducting behavior of MgB_2_ intercalated muscovite. Superconductivity in MgB_2_ is attributed to the coupling of *E*_2g_ phonons and the σ_px,y_ band, which exhibits planar orientations in space.^23^ From an electronic structure perspective, the σ_px,y_ band is a critical source of superconductivity, primarily due to its significant contribution from the boron–boron bonding, which constitutes a substantial portion of the hybridized orbitals. This interaction enhances the coupling constant, stabilizing the σ_px,y_ band and enabling the maintenance of superconductivity at higher temperatures.^24^ Applying pressure affects the vibrational state of *E*_2g_ phonons, specifically causing an increase in their vibrational frequency and hardening the phonon mode. This increase results in a decreased coupling constant, as the system becomes less inclined to couple with the σ_px,y_ band. Additionally, pressure induces a shift in the Fermi level electrons associated with the σ_px,y_ band, leading these electrons to vacate the state. This shift reduces electron pairs (Cooper pairs),^25^ decreasing the coupling constant. This interaction points out the sensitivity of the superconducting state of MgB_2_ to external mechanical stresses, emphasizing the crucial role of phonon–electron coupling dynamics in determining its superconducting properties. We used Raman spectroscopy to verify the bending effect on the MgB_2_–muscovite system, which is sensitive to structural change. The pristine state and bending measurements showed a peak shift from 525.2 cm^–1^ (black) to 532.0 cm^–1^ (red), with the peak returning to 525.2 cm^–1^ (blue) upon release, indicating the mechanical sensitivity of the system to external strain (Figure 4A) with an estimate of 0.62 GPa.^24^

In this section, we discuss two main effects: the intercalation effect and the bending effect on the MgB_2_–muscovite systems. In principle, applying external stress to MgB_2_ changes its superconducting critical temperature *T*_c_. The induced pressure discussed in this paragraph is primarily governed by the following relationship (eq1), where *T*_c_ represents the critical temperature of MgB_2_ and *P* denotes the applied pressure^15^
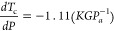
1

To avoid the flux pinning effect,^26^ we employed zero-field cooling (ZFC) measurements using a superconducting quantum interference device (SQUID). Through temperature-dependent measurements, we identified a transition point where the magnetization became negative, as indicated by the second-order differential. This observation confirms the Meissner effect, which is a characteristic behavior of superconductivity. To calibrate the baseline, bare muscovite and pure MgB_2_ powder were first conducted for the ZFC measurements. In Figure 4B, the magnetization of muscovite increases with decreasing temperature, exhibiting typical paramagnetic behavior. Conversely, commercial MgB_2_ powder presents a sharp transition to negative magnetization at 38.94 K, indicating a high-quality superconducting source (Figure 4C). Further MgB_2_–muscovite composite ZFC measurements revealed a transition temperature at 38.55 K, suggesting an internal pressure of approximately 0.35 GPa and the Meissner volume (*%V*_SC_) fraction of the MgB_2_–muscovite composite is about 0.014%. The detailed calculation process has been discussed in eqs S1–S3. Bending measurement resulted in a further decrease in *T*_c_ to 37.25 K, corresponding to an internal pressure of 1.17 GPa, closely matching the initial estimate of the muscovite bending stress at 1.72 GPa. Upon bending release, *T*_*c*_ increased to 38.28 K, 0.27 K lower than that under the flat condition (38.55 K), attributable to thermal stress stored in the composite during the ZFC process. The thermal stress,^27^ from 300 to 10 K, is around 0.20 GPa, which can induce a 0.22 K decrease in the system, closely aligning with the observed change (0.27 K). The onset temperature of MgB_2_/muscovite is less than that of the powder due to the strain effect. Under pressure, the frequency of the *E*_2g_ vibrational mode significantly increases. The change in vibrational frequency reduces coupling, lowering the coupling constant and consequently causing a decrease in *T*_c_. Under external stress, the charge carriers (electron holes) in *p*_x,y_ transfer to *p*_z_. Since *p*_x,y_ is one of the hybrid orbitals contributing to superconductivity, the loss of carriers in *p*_x,y_ will decrease *T*_c_. These findings demonstrate (Table 1) a high degree of consistency among the effects of intercalation, bending, and thermal stress on the induced changes in *T*_c_ and the stresses endured, demonstrating muscovite as a stable platform for high-pressure physics exploration.

**Table 1:** Summary of Pressure Induced Through Different Types of Stress

Stress type (approach)	Pressure
Intercalation effect (Δ*T*_c_)	∼0.35 GPa
Bending effect (Δ*T*_c_)	∼1.17 GPa
Bending effect (Raman shift)	∼0.62 GPa

In conclusion, this research introduces a novel method for applying stress to the superconducting MgB_2_ intercalated muscovite system. This method allows for estimating applied stress based on observed changes in the Raman shift. Additionally, through the Meissner effect, the change in *T*_c_ can verify the magnitudes of both static and dynamic stress. This intercalation method offers a scalable and customizable approach that preserves various material properties. This method provides a unique platform for fundamental research and serves as a simpler alternative to the DAC system, facilitating the exploration of material properties in reduced dimensions. Moreover, the ability to study these new nanocomposites under ambient conditions overcomes a critical barrier associated with high-pressure research, paving the way for practical applications and significant advancements in the study of intercalation in layered composite materials.

## Methods

### Sample Preparation

The muscovite substrate was sealed into the quartz tube with MgB_2_ powder (>99%, Thermo Scientific Corp.) in a 5 × 10^–3^ torr atmosphere. The sealed quartz tube underwent a two-step gas-phase intercalation process, starting with annealing at 800 °C to initiate nuclei formation and cooling to 600 °C for crystal growth (Figure S2A). Process optimization mainly focuses on nucleation quantity (T1) and crystal growth time (T2). The corresponding results have been discussed in Figure S2B,C, and Table S1 shows the MgB_2_ content and *T*_c_.

### Structural Analysis

The distribution and orientation of the MgB_2_ crystal structure were presented through nanobeam X-ray Laue diffraction at BL 21A1 of the Taiwan Photon Source at the National Synchrotron Radiation Research Center, Taiwan. A focused white/mono X-ray with a dimension of 90 × 90 nm^2^ was used to determine the crystal structure and perform 2D mapping with a high-precision scanning stage (SmarAct). Reflected nanodiffraction patterns were collected using a high-sensitivity hybrid pixel array detector (Pilatus3-X-6M), providing an angular resolution better than 0.018°. The TEM specimen was prepared using the focused ion beam technique via Matek Corp. and examined using a JEOL JEM-F200 microscope.

### Spatial-Resolution Raman Measurements

Spatial-resolution Raman spectroscopy (WITec, alpha300 R) was conducted using a 532 nm laser to distinguish the MgB_2_ intercalant from the potential MgO byproduct. The WITec alpha300 R is a research-grade microscope with an ultrahigh-throughput spectrometer for Raman imaging. The laser power used for the measurements was 20 mW, and the setup configuration was backscattering. The lateral resolution is physically limited to approximately 200 nm.

### Chemical Stoichiometry

The SEM measurements were performed by a JSM-IT800. Before the sample was placed into the SEM, it was cut into a 3 × 3 mm^2^ size. A thin carbon layer was coated on it to avoid the charging effect during the measurement. The acceleration voltage of the electron gun was 10 kV, and an upper-in-lens detector was applied. The EMPA measurements were conducted using a JXA-IHP200F, and the sample preparation was the same as that for the SEM specimen. The elemental resolution of EMPA is 5 to 10 eV, which is larger than that of EDS (130 to 140 eV) and capable of analyzing elements from Be (*Z* = 4) to U (*Z* = 92).

### Magnetic Measurements

The ZFC measurements were characterized using a Quantum Design MPMS or PPMS-VSM. The sample was cut into 3 × 3 mm^2^ pieces and mounted on a quartz tube. After that, the sample was cooled at a rate of 2 K/min. To avoid the remanence of the superconducting magnet, we applied an in-plane field of 100 Oe for the ZFC measurement.

### Bending Tests

Magnetic temperature-dependent measurements were made by taping the sample onto the homemade molds and binding them with Kapton tape.

## Supporting Information Available

Additional structural information of XND, the optimization of %*V*_sc_, and the calculation of %*V*_sc_ (PDF)
