# Supplementary material for: Superconductive MgB2 Intercalated Muscovite with Dynamically Tunable Stress
Source: ACS Omega. 2024 Sep 10;9(38):39856–62. doi: 10.1021/acsomega.4c05303 (PMC11425818; doi:10.1021/acsomega.4c05303)
Supplement: Supplementary file 1 — ao4c05303_si_001.pdf [file ao4c05303_si_001.pdf]

Superconductive MgB_2 Intercalated Muscovite with Dynamically Tunable Stress

Shu-Hua Kuo[1], Yi-Cheng Chen[1], Yu-Chieh Wang[2], Wan-Zhen Hsieh[3], Ching-Yu Chiang[3], Cheng-Maw Cheng[3], Lu-Hsing Chen[4], Kuo-Ping Chen[4], Yu-Hao Tu[5], Jiunn-Yuan Lin[2], Ying-Hao Chu[15*]*

[1]Department of Materials Science and Engineering, National Tsing Hua University, Hsinchu, 300044, Taiwan

[2]Institute of Physics, National Yang Ming Chiao Tung University, Hsinchu 300093, Taiwan

[3]National Synchrotron Radiation Research Center, Hsinchu, 300092, Taiwan

[4]Institute of Photonics Technologies, National Tsing Hua University; Hsinchu, 300044, Taiwan

[5]College of Semiconductor Research, National Tsing Hua University; Hsinchu, 300044, Taiwan

KEYWORDS

reduced dimension, intercalation, muscovite, composites, superconductivity

The Supporting Information is available free of charge at ACS Omega.

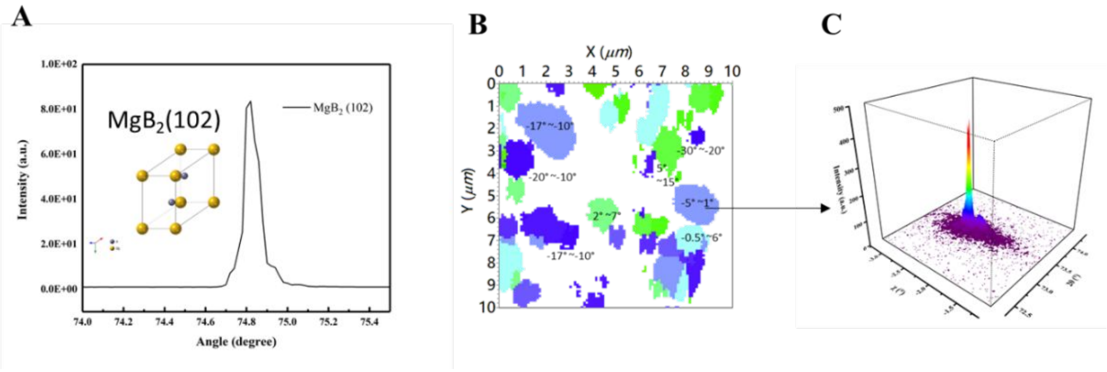

Figure S1 Structural information of XND. (A) The diffraction peak of MgB_2(102). (B) The distribution of χ angle of different MgB_2 crystals. (C) The intensity distribution as a function of the χ angle in a selected MgB_2 crystal

The thermal process is critical to achieving continuity, as Figure S2A shows. Process optimization (Figure S2B) mainly focuses on nucleation quantity (T1) and crystal growth time (T2). At T1, the heat treatment process can loosen the van der Waals forces between the layers of muscovite, allowing MgB_2 vapor to more effectively intercalate. In Figure S2C, the projection of *% Vsc* (blue spot) shows that with increasing T2, the Meissner volume rises accordingly. Therefore, we infer that to achieve a continuous MgB_2 layer, increasing the heat treatment time (T1) would be a solution. However, the superconductive properties depend on the T2 process. Thus, achieving a continuous functional MgB_2 layer requires increasing T1 and T2 heating times.

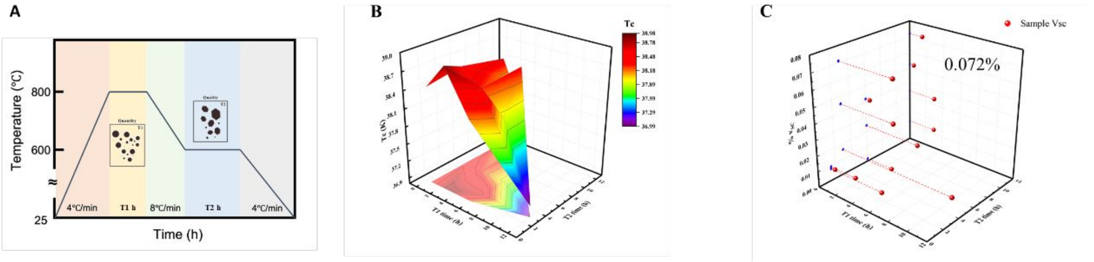

Figure S2 Optimization for $\%V_{SC}$ and T_C. (A) The temperature-time curve of intercalation process. (B) T_C and (C) $\%V_{SC}$ with different annealing times T1 and T2.

#	T1	T2	%Vsc	Tc
1	2	12	0.0720	38.1
2	6	3	0.0712	38.3
3	1	12	0.0520	38.4
4	6	3	0.0455	39.0
5	1	6	0.0418	38.6
6	3	12	0.0340	38.9
7	6	6	0.0244	37.9
8	12	3	0.0163	37.0
9	3	12	0.0127	37.8
10	3	2	0.0091	38.9
11	1	2	0.0084	38.5
12	6	2	0.0077	38.2
13	3	6	0.0017	38.3

Table S1 Table of parameters for sample 1 to sample 13.

We have listed the calculation process in the supporting information to make it more clearly. In this VSM measurement, we applied an external magnetic field (H) of 500 Oe. The difference (dm) between the lowest magnetic moment value (-1.41×10^{-5} emu) in the MT data and the magnetic moment value at the T_c (8.71×10^{-7} emu). We assume all the magnetic moment is contributed by the MgB_2, which constitutes the superconductive volume(d $V = V_{SC}$). Finally, we obtained an effective superconducting volume ratio of 0.0418%.

$$B = B_a + \mu_0 M = 0 \text{ ------Eq. S1}$$

$$M = -\frac{1}{\mu_0} B_\mathrm{a} = \frac{\mathrm{d}m}{\mathrm{d}V} \text{ ------Eq. S2}$$

$$\%V_{sc} = \frac{V_{sc}}{V_s} \text{ ------Eq. S3}$$

B_a: applied magnetic field

μ_0: permeability of vacuum

M: magnetization

m: magnetic dipole moment

V: volume

V_{SC}: superconductive volume

V_S: sample volume

$\%V_{SC}$: Meissner volume
